# Organogenic nodule development in hop (*Humulus lupulus *L.): Transcript and metabolic responses

**DOI:** 10.1186/1471-2164-9-445

**Published:** 2008-09-29

**Authors:** Ana M Fortes, Filipa Santos, Young H Choi, Marta S Silva, Andreia Figueiredo, Lisete Sousa, Fernando Pessoa, Bartolomeu A Santos, Mónica Sebastiana, Klaus Palme, Rui Malhó, Rob Verpoorte, Maria S Pais

**Affiliations:** 1 ICAT, FCUL, University of Lisbon, Campo Grande, 1749-016 Lisbon, Portugal; 2 Division of Pharmacognosy, Section Metabolomics, Institute Biology Leiden, Leiden, the Netherlands; 3 Department of Chemistry and Biochemistry, FCUL, Lisbon, Portugal; 4 Department of Statistics and Operational Research, CEAUL (Centro de Estatística e Aplicações da UL), FCUL, Lisbon, Portugal; 5 Institute for Biology II/Botany, Albert-Ludwig's University, Freiburg, Germany; 6 Dep. Micologia, University Federal de Pernambuco, Av. Prof. Nelson Chaves s/n, Cidade University, 50670-420, Recife, PE, Brazil

## Abstract

**Background:**

Hop (*Humulus lupulus *L.) is an economically important plant forming organogenic nodules which can be used for genetic transformation and micropropagation. We are interested in the mechanisms underlying reprogramming of cells through stress and hormone treatments.

**Results:**

An integrated molecular and metabolomic approach was used to investigate global gene expression and metabolic responses during development of hop's organogenic nodules.

Transcript profiling using a 3,324-cDNA clone array revealed differential regulation of 133 unigenes, classified into 11 functional categories. Several pathways seem to be determinant in organogenic nodule formation, namely defense and stress response, sugar and lipid metabolism, synthesis of secondary metabolites and hormone signaling. Metabolic profiling using ^1^H NMR spectroscopy associated to two-dimensional techniques showed the importance of metabolites related to oxidative stress response, lipid and sugar metabolism and secondary metabolism in organogenic nodule formation.

**Conclusion:**

The expression profile of genes pivotal for energy metabolism, together with metabolites profile, suggested that these morphogenic structures gain energy through a heterotrophic, transport-dependent and sugar-degrading anaerobic metabolism. Polyamines and auxins are likely to be involved in the regulation of expression of many genes related to organogenic nodule formation. These results represent substantial progress toward a better understanding of this complex developmental program and reveal novel information regarding morphogenesis in plants.

## Background

Somatic embryogenesis is widely used to propagate different species such as coffee, mangos and roses. It helps fast breeding of new varieties, the production of hybrid seedlings and reduction of heterogeneous transmission of genetic traits to the progeny. This capacity in plants to form embryos and morphogenic structures from a diverse set of tissues (totipotency) is integral to plant biotechnology and these techniques and protocols have been incorporated into many breeding programs. However, the mechanisms that control these processes remain far from being understood. Such insights would not only shed light on how cell fates become fixed during development, and how plants manage to retain such plasticity, but also provide tools for numerous applications of *in vitro *plant biology, such as propagation of many recalcitrant crop plants.

In recent years, progress has been made regarding the molecular bases underlying both *in vitro *and *in vivo *plant morphogenesis [1,2]. Organogenic nodular structures have been studied in several plant species and, as somatic embryos, they constitute a morphogenic pathway useful for regeneration strategies, automated micropropagation, and genetic transformation for desirable traits [3]. Organogenic nodules form a cohesive unit able to undergo cell and tissue differentiation, but unlike embryos those structures show no polarity and can regenerate shoot buds all over their surface [3]. Moreover, organogenic nodules show multiple vascularization centers around which nodulation can occur and form small "daughter nodules". This separation process seems to be initiated by the formation of a necrosis layer at the future place of nodule separation [3].

Organogenic nodule formation has been previously described in hop (*Humulus lupulus *L.)[4], an economically important plant known for the production of acid resins and essential oils used in brewing and for medicinal properties [4,5]. For these reasons, the genomic resources for hop have been recently expanded [5]. In previous studies, we reported an important role for starch accumulation, expression of lipoxygenases and extracellular signal-regulated kinases as well as synthesis of reactive oxygen species, jasmonic acid and polyamines in organogenic nodule formation [4,6-9]. These data suggested that organogenic nodule formation results from a stress response to both wounding and *in vitro *conditions, involving a strong accumulation/mobilization of carbohydrates and lipids. In soybean, changes in mRNA abundance of genes characteristic of oxidative stress and cell division suggested that arrangement of cells into organized structures might depend on a tight control between cell proliferation and cell death [1]. Cell competence seems to be associated with a particular metabolic cell-state, which enables, under stress conditions, to switch on defense mechanisms that promote morphogenesis. Like in other plants, reactive oxygen species are not only stress signal molecules, but important intrinsic signals which together with sugar and hormones affect somatic embryo formation and seedling development [10-12].

Transcriptomic studies have unraveled the molecular details underlying developmental processes such as fruit ripening [13], formation of symbiotic nodules [14] and somatic embryogenesis [1,15]. Combined transcript and metabolite studies may have the potential to elucidate gene functions and networks in these processes. Metabolite profiling in conjunction with selective mRNA and physiological profiling has been used to characterize Arabidopsis seeds throughout development and germination [16]. Nuclear Magnetic Resonance (NMR) spectroscopy detects a broad range of metabolic groups; it constitutes a fast and accurate tool for discriminating between groups of related samples and provides a chemical "snapshot" of an organism's metabolic state [17-19]. In particular, ^1^H NMR, yields a comprehensive fingerprint of all hydrogen-attached extractable metabolites. It can also provide direct structural information regarding individual metabolites in the mixture, particularly when two dimensional techniques are applied [20,21].

Here we used transcriptional and metabolic profiling to study organogenic nodule development in hop. This integrated approach aimed at dissecting the genetic control of plant cell totipotency in *in vitro *culture using hop organogenic nodules as a model. Integrated analysis of the metabolome and transcriptome of these morphogenic structures indicates that the control of different stages of plant morphogenesis depends on integration of defense and stress responses, hormone synthesis and changes in generation of energy, sugar and lipid metabolism. Our data allowed a detailed assessment of the different developmental stages, and together with our previous molecular and cellular studies provide an integrated comprehensive model of hop organogenesis.

## Results

### Selection of stages during hop organogenic nodule development

To investigate the spatial-temporal sequence of events that underlies competence acquisition for organogenesis four main stages were selected: (i) internodes at the time of excision from the parent plant (T0); (ii) internodes grown for 24 h (T24h); (iii) internodes grown for 15 days on culture medium in which several prenodular structures are formed inside the *calli *(T15d); and (iv) nodule forming tissue after 28 days of culture (T28d). Additionally a control was included corresponding to 28 days of culture without hormones, lacking nodule formation and plantlet regeneration abilities, in order to identify genes specifically involved in morphogenesis (T28dWH).

**Figure 1:**
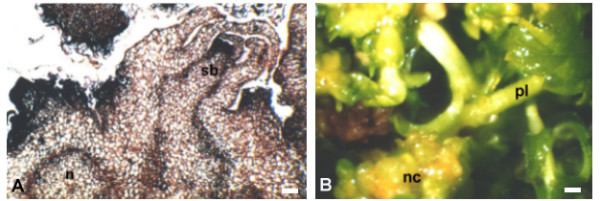
**Organogenic nodule formation in hop**. **A **Detail of a transversal section of a nodule after 45 days in culture showing one shoot bud (sb) connected to the nodular vascular bundles. Material was previously embedded in paraffin wax. **B**. Nodule cluster (nc) formed after 45 days in culture and showing several shoot buds and plantlets (pl). Bars in A = 150 μm, in B = 800 μm.

Organogenic nodule formation is a morphogenic process that shares features with somatic embryogenesis though in the former no shoot/root pole is established and plantlet regeneration can occur from different peripheral regions of nodules (Figure 1A–B) [4]. Previous studies have shown that the organogenesis-determining period (when cells are determined to form nodules) occurs in between 15 and 25 days of culture corresponding to prenodular and first nodular stages. When nodules are fully developed they are surrounded by layers of elongated, highly vacuolated cells that may degenerate when nodules start differentiating plantlets. Plantlet regeneration from organogenic nodules can be observed after 45 days of culture. However, organogenic nodules after 28 days of culture are already determined to undergo plantlet regeneration, since they no longer require exogenous supply of hormones [4]. Explants cultured in medium without hormones can develop incipient prenodular structures with vascular tissue but not nodules, thus lacking morphogenic potential [4].

### Transcriptional profiling during organogenic nodule induction and formation

#### cDNA Microarray data

A cDNA library representing the stages of hop nodule development was constructed, 3308 ESTs were isolated and spotted onto glass chips. In addition to these, 16 cDNAs previously cloned by an RT-PCR approach were also spotted (Methods). Sequencing of 80 clones showed that the redundancy of the cDNA library could be estimated to be about 45%. Changes in gene expression pattern during formation of organogenic nodules were analyzed by comparing transcript abundance of samples from sequential developmental stages to a common reference (T0). In a subsequent set of experiments organogenic nodules cultured for 28 days on medium with hormones (T28d) were compared to samples cultured also for 28 days but in medium without hormones (T28dWH).

Four hybridizations were carried out per time point to obtain the expression value for each gene (fold change of normalized signals), except for the time point corresponding to 24 h. For the T24h versus T0 experiment only two hybridizations were carried out in order to evaluate the behaviour of the differentially expressed genes obtained for the other time points at an early stage as 24 h (see Methods). Ratios (fold change of normalized signals) of the clones found within the same contig were averaged and counted as one gene (contigs/unigenes were named according to the lowest clone number belonging to that contig).

Transcription of 133 unigenes was significantly up- or down- regulated during induction and development of organogenic nodules (Table 1). Similarities were checked using public databases (National Center for Biotechnology Information-NCBI and The Arabidopsis Information Resource-TAIR). Among these were sequences displaying similarity to unknown proteins or expected to be newly identified genes (12.03% of the contigs). To extract biological knowledge when examining the changes in gene function that were occurring during morphogenesis, functional categories for the hop genes were identified based upon the GO annotations regarding the Biological Process, a systemized annotation vocabulary describing biological function defined by the Arabidopsis Information Resource (TAIR, using Gene Ontologies, see Additional file 1). For all the differentially expressed hop genes, the Arabidopsis gene with the best sequence similarity based on BLASTX analysis was selected (BLAST scores below a cut-off criterion of 10^-7 ^for BLAST e-values); and TAIR GO associated with biological process for that Arabidopsis gene assigned to the hop gene.

**Table 1:** Genes differentially expressed during organogenic nodule formation in hop.

**GenBank Acc. No**.	**E value**	**Annotation**	**24 h vs 0**	**15d vs 0**	**28d vs 0**	**28d vs 28dWH**	**Clone ID/contig**
		***Defense response, response to stress, response to chemical stimulus, abiotic and biotic stimuli***					

ES437675	1,00E-81	cationic peroxidase [*Nelumbo nucifera*]	-3.81	-1.44		**2.01**	Hlct1
ES437719	9,00E-119	peroxidase [*Vigna angularis*]	2.73	1.53	1.79	**2.03**	Hl4206
ES437713	2,00E-87	peroxidase [*Populus trichocarpa*]		**2.16**	1.66		Hl4578
ES437767	2,00E-13	SAG21 (Senescence-Associated Gene 21) [*Arabidopsis thaliana*]	1.87	**2.09**	1.87	**-1.91**	Hl4571
ES437753	4,00E-77	Senescence-associated [*Medicago truncatula*]	4.06	**4.32**	**3.46**		Hl1471
ES437674	0.0	*M. domestica *ribulose-1.5-bisphosphate carboxylase/oxygenase activase	**-13.29**	**-5.38**	**-2.97**		Hlct810
ES437696	1,00E-64	major allergen Pru p 1 [*Prunus persica*]	**6.05**	**3.5**	1.95		Hl1695
ES437747	2,00E-48	major allergen Pru p 1 [*Prunus persica*]	**5.94**	**3.74**	**2.56**		Hlct313
ES437764	2,00E-140	*Solanum tuberosum *clone 054G03 Hsp90-2-like mRNA	2.02	**2.81**	**3.1**		Hlct1560
ES437687	2,00E-23	putative gamma-thionin [*Castanea sativa*]	5.21	1.55		**-1.87**	Hl1925
ES437729	5,00E-20	mandelonitrile lyase [*Arabidopsis thaliana*]		-1.46	NA	**4.06**	Hl2222
ES437733	3,00E-152	Thiazole biosynthetic enzyme, chloroplast precursor	-3.34	**-3.29**	**-2.49**	**-2.35**	Hl3328
ES437789	2,00E-162	*Humulus lupulus *chitinase	1.74	**2.52**	2.38		Hlct1044
ES437688	1,00E-65	glutathione S-transferase GST 12 [*Glycine max*]	4.2	**2.63**	**2.62**		Hl1525
ES437709	1,00E-16	early flowering 3 [*Mesembryanthemum crystallinum*]	1.56	1.82	**2.64**		Hl3446
ES437740	1,00E-12	nematode responsive protein [*Arabidopsis thaliana*]	2.64	**4.57**	**3.12**		Hl4497
EF624245	*6,00E-124*	*putative phenylalanine ammonia-lyase [Rhizophora mangle]*	1.95	1.56	*NA*		*Hlpal*
ES437730	3,00E-32	*Populus tremula *× *Populus tremuloides *aux/IAA protein	-1.41	-1.54	**-2.5**	**2.55**	Hl4491

		***Macromolecule metabolic processes***					

ES437720	6,00E-10	*Solanum tuberosum *extensin	1.97	**7.03**	**4.44**		Hlct117
AY795910	0.0	*Humulus lupulus beta-1.3-glucanase*	3.81	***3.37***	***3.28***		*Hlgluc5*
EF593131	2,00E-79	Pectin methylesterase 1 [*Pyrus communis*]		1.65			Hl52
ES437689	4,00E-121	*Populus tremula *xyloglucan endotransglycosylase/hydrolase precursor XTH-30	3.48	**5.3**	**6.87**		Hl3465
ES437757	0.0	*Nicotiana tabacum *ZIP	-5.77	**-3.4**	NA	NA	Hl4167
EF624249	*5,00E-158*	*sucrose synthase [Glycine max]*	3.01	***1.9***	1.5		*Hlsuc*

		***Cellular metabolic processes***					

ES437727	1,00E-56	RNA binding [*Arabidopsis thaliana*]	2.15	**2.19**	**2.32**		Hl417
ES437761	4,00E-161	Avr9/Cf-9 induced kinase 1 [*Nicotiana tabacum*]	2.46	**2.53**	**3.17**		Hl3459
ES437769	6,00E-20	Plant lipid transfer protein/Par allergen [*Medicago truncatula*]	2.66	**2.71**	1.76		Hl3157
ES437798	1,00E-156	*Hevea brasiliensis *latex plastidic aldolase-like protein	**-9.51**	**-4.32**	**-2.85**		Hlct77
ES437772	1,00E-94	ribonuclease/transcriptional repressor [*Arabidopsis thaliana*]		-1.44	**-2.67**	NA	Hl4319
ES437711	8,00E-81	pfkB-type carbohydrate kinase family protein, putative expressed [*Oryza sativa*]	3.54	**2.49**	**2.8**		Hl3980
ES437721	0.0	putative dTDP-glucose 4-6-dehydratase [*Arabidopsis thaliana*]	NA	**2.07**	**2.23**		Hl4620
ES437736	0.0	*Arabidopsis thaliana *mRNA for glyceraldehyde-3-phosphate dehydrogenase C subunit	2.31	**2.5**	1.53		Hlct23
ES437788	2,00E-152	fructose-bisphosphate aldolase-like protein [*Solanum tuberosum*]	NA	**1.91**		**-1.87**	Hlct3973
ES437680	6,00E-63	glyceraldehyde-3-phosphate dehydrogenase A subunit, photosynthetic isoform [*Glycine max*]	**-10.78**	**-5.17**			Hl263
ES437780	6,00E-83	glycerophosphodiesterase-like protein [*Nicotiana tabacum*]	-1.6		-1.58	**2.65**	Hl3510
ES437786	1,00E-95	epoxide hydrolase/hydrolase [*Arabidopsis thaliana*]			NA	**3.18**	Hl1072
ES437692	0.0	*C. blumei *kinetoplast met gene for cobalamine-independent methionine synthase	3.56	**2.13**	1.45		Hlct4592
ES437722	4,00E-09	S-adenosylmethionine decarboxylase [Malus × domestica]	NA	NA	**2.6**	NA	Hl3196
ES437681	0.0	*Elaeagnus umbellata *S-adenosyl-L-methionine synthetase (SAMS2) mRNA	1.55	**2.35**	1.48	-1.68	Hlct214
ES437707	1,00E-80	*Vitis vinifera *3-deoxy-D-arabino-heptulosonate 7-phosphate synthase	3.25	**2.31**	NA		Hl1812
ES437758	3,00E-85	*Capsicum annuum *nucleoside diphosphate kinase	4.72	**5.1**	**5.03**		Hl1543
ES437763	3,00E-82	MAP kinase-like protein [*Gossypium hirsutum*]	-1.89	-1.66	-2.08	**2.48**	Hl4516
ES437718	5,00E-58	Protein kinase; Type I EGF [*Medicago truncatula*]	-1.46	-1.62	**-4.38**	**7.51**	Hl4559
ES437766	5,00E-77	Putative phosphatase 2A inhibitor [*Arabidopsis thaliana*]	-1.68	-1.92	-1.67	**2.35**	Hl2273

		***Primary metabolic process***					

ES437723	5,00E-11	*Corylus avellana *lipid transfer protein precursor			1.95	**1.99**	Hl2898
ES437779	1,00*E*-14	*Lipoxygenase [Medicago truncatula]*	3.35	***2.42***			*Hllox6*
ES437705	2,00E-31	Lipoxygenase [*Nicotiana attenuata*]			**-3.26**	**4**	Hl2195

		***Transcription and DNA and RNA metabolism***					

ES437679	2,00E-32	ZFP4 (ZINC FINGER PROTEIN 4); nucleic acid binding/transcription factor/zinc ion binding [*Arabidopsis thaliana*]	3.83	**2.99**	**3.35**		Hl1813
ES437796	1,00E-43	histone H2B1 [*Gossypium hirsutum*]	2.66	**2.57**	**2.36**		Hl2418
ES437677	2,00E-62	*A. thaliana *histone H4 gene	3.76	**1.94**	1.73	**2.03**	Hlct2847

		***Signal transduction***					

ES437693	5,00E-70	*Medicago truncatula *small G-protein ROP9	**6.54**	**7.27**	**6.23**		Hl1506
ES437706	3,00E-118	*Populus tomentosa *calmodulin	1.71	**2.09**	1.4		Hl2815

		***Cellular component organization and biogenesis***					

ES437700	1,00E-39	*Gossypium hirsutum *RAC-like G-protein Rac1	2.04	**2.02**	1.98		Hl1537
ES437797	1,00E-113	*Camellia sinensis *alpha tubulin 1 (Tua1)			**-5.7**	**7.36**	Hl2210
ES437690	6,00E-12	*Arabidopsis thaliana *POK (POKY POLLEN TUBE)	**-8.51**	**-2.97**	-1.62	**1.99**	Hl3610

		***Protein metabolism***					

ES437748	8,00E-36	ribosome inactivating protein Euserratin 2 precursor [*Euphorbia serrata*]	1.7		1.71	**2.86**	Hl3627
ES437712	6,00E-76	*A. thaliana *structural constituent of ribosome (AT5G28060)	2.82	**2**	**2.3**		Hl1643
ES437716	5,00E-113	*Cicer arietinum *mRNA for ribosomal protein RL5 (rl5 gene)	3.63	**5.31**	**5.35**		Hl1520
ES437765	3,00E-57	structural constituent of ribosome [*Arabidopsis thaliana*]			-1.44	**3.03**	Hl3805
ES437756	5,00E-41	*Triticum aestivum *ribosomal protein L39	2.8	**2.54**	**2.2**		Hl4115
ES437773	2,00E-55	40S RIBOSOMAL PROTEIN S20 homolog [*Arabidopsis thaliana*]	2.85	NA	**2.29**	NA	Hl1619
ES437744	6,00E-56	ribosomal protein L30 [*Lupinus luteus*]	3.72	**3.71**	**2.83**		Hlct1532
ES437739	3,00E-26	*G. hirsutum *mRNA for ribosomal protein 41, large subunit (RL41)	4.22	**2.17**	1.57		Hlct1769
ES437701	7,00E-15	putative subtilisin-like serine proteinase [*Arabidopsis thaliana*]	1.45	1.88	**2.2**		Hl1547
ES437775	8,00E-18	*Trifolium pratense *RNA for putative zinc dependent protease	NA	NA	**2.63**	NA	Hl1923
ES437778	3,00E-21	protein binding/ubiquitin-protein ligase/zinc ion binding [*Arabidopsis thaliana*]	NA	1.88	**2.17**		Hl1495
ES437685	6,00E-25	ATP binding/protein binding [*Arabidopsis thaliana*]	-1.47	-1.62	**-3.24**	**3.09**	Hl2061

		***Photosynthesis and carbon utilization***					

ES437710	7,00E-165	oxygen evolving complex 33 kDa photosystem II protein [*Nicotiana tabacum*]	**-10.45**	**-4.95**	**-2.56**		Hlct484
ES437702	5,00E-88	*Arachis hypogaea *photosystem I psaH protein	**-7.75**	**-4.86**	**-2.28**		Hlct829
ES437794	2,00E-46	photosystem I reaction center subunit × psaK [*Nicotiana tabacum*]	**-6.29**	NA	**-2.68**	NA	Hlct526
ES437741	0.0	*Glycine max *cv. Dare photosystem II type I chlorophyll a/b-binding protein (lhcb1*7) gene	**-9.9**	**-6.33**	**-2.84**	1.66	Hlct54
ES437745	1,00E-144	chlorophyll ab binding protein [*Gossypium hirsutum*], light harvesting complex	**-11.69**	**-5.89**	**-2,43**	1.86	Hlct362
ES437704	1,00E-95	photosystem II 23 kDa polypeptide [*Nicotiana tabacum*]	**-7.41**	**-4.34**	-1.89		Hl2435
ES437703	2,00E-131	chlorophyll a/b binding protein [*Solanum tuberosum*]	**-6.91**	**-5.95**	**-2.97**		Hlct1969
ES437759	2,00E-07	LHCII type I chlorophyll a/b binding protein [*Vigna radiata*]	**-18.13**	**-8.33**	**-3.71**	1.78	Hl240
ES437783	6,00E-118	chloroplast pigment-binding protein CP26 [*Nicotiana tabacum*]	-3.02	**-3.13**	**-3.48**	**2.92**	Hl4149
ES437793	1,00E-86	PSI type III chlorophyll a/b-binding protein [*Arabidopsis thaliana*]	-4.96	**-4.08**	**-2.35**		Hl2281
ES437755	3,00E-118	LHCII type III chlorophyll a/b binding protein [*Vigna radiata*]	NA	**-7.85**	**-3.04**		Hl3332
ES437760	5,00E-79	putative chloroplast chlorophyll a/b-binding protein [*Carya cathayensis*]	**-7.19**	**-4.82**	**-2.67**		Hl1396
ES437673	7,00E-85	small subunit ribulose-1.5-bisphosphate carboxylase/oxygenase [*Fagus crenata*]	**-13.36**	**-6.44**	**-2.49**		Hlct105
ES437715	1,00E-128	chlorophyll a/b-binding protein CP24 precursor [*Vigna radiata*]	**-7.11**	**-5.46**	**-2.79**		Hlct1168
ES437762	1,00E-130	chlorophyll a/b-binding protein [Solanum lycopersicum]	**-9.86**	**-6.57**	**-2.75**		Hlct2446
ES437697	1,00E-74	Potato mRNA for light inducible tissue-specific ST-LS1 gene	**-11.42**	**-4.33**	**-2.33**		Hlct30
ES437781	4,00E-07	putative photosystem I reaction centre PSI-D subunit precursor [*Solanum tuberosum*]	-1.78	-1.85	-1.74	**3.03**	Hl3804
ES437771	7,00E-68	subunit of photosystem I [*Cucumis sativus*]	-4.84	**-4.02**	**-2.08**		Hlct2254

		***Generation of precursor metabolites and energy***					

ES437678	7,00E-24	putative photosystem I reaction center subunit IV [*Arabidopsis thaliana*]	-4.51	**-3.63**	-1.85		Hl4247
ES437751	8,00E-87	*Pachysandra terminalis *glycolate oxidase	**-7.94**	**-3.22**	**-2.46**		Hl1696
ES437768	2,00E-39	putative steroid binding protein [*Arabidopsis thaliana*]	-1.9			**-1.87**	Hl1863
ES437752	3,00E-62	F1-ATP synthase delta subunit [*Ipomoea batatas*]	NA	**2.35**	**2.87**		Hl1512
ES437695	2,00E-138	*Gossypium hirsutum *vacuolar H+-ATPase subunit B	2.52	**2.82**	**2.8**		Hl3429
ES437683	8,00E-94	vacuolar-type H+-ATPase (v-ATPase) subunit D [*Arabidopsis thaliana*]	1.61	1.85	**2.33**		Hl3612

		***Secondary metabolic process***					

ES437792	7,00E-72	NADPH-protochlorophyllide oxidoreductase [*Cucumis sativus*]	-4.21	**-3.95**	**-2.26**		Hlct395
ES437754	2,00E-173	Malus × domestica cinnamic acid hydroxylase (C4H1)	2.03	**2.24**	1.67		Hlct225
ES437670	7,00E-93	iron ion binding/isopenicillin-N synthase/flavonol synthase [*Arabidopsis thaliana*]	2.44	**5.27**	**2.33**		Hl3668

		***Other classes***					

ES437676	1,00E-55	protein transporter [*Arabidopsis thaliana*]	-1.47	-1.84	**-2.25**	**4.29**	Hl1064
ES437750	3,00E-22	*Gossypium hirsutum *hybrid proline-rich protein 2		**2.43**			Hlct2443
ES437749	1,00E-08	metallothionein 1a [*Populus balsamifera *subsp. trichocarpa × *Populus deltoides*]	1.74	**2.61**	**2.13**		Hlct1310
ES437717	7,00E-54	*Fagus sylvatica *glycine-rich protein 2	2.55	**2.08**		NA	Hl1692

		***Unknown function***					

ES437698	3,00E-16	nectarin IV [*Nicotiana langsdorffii *× *Nicotiana sanderae*], xyloglucan-specific fungal endoglucanase-inhibitor		-1.46	-1.79	**2.69**	Hl3829
ES437708	2,00E-67	Stress protein DDR48, related [*Medicago truncatula*]	1.65	**2.22**	**2.9**		Hl3456
ES437737	6,00E-78	hypothetical protein OsJ_005893 [*Oryza sativa*], sodium/calcium exchanger protein	2.11	**3.17**	**3.52**		Hlct2641
ES437724	2,00E-25	putative type-1 pathogenesis-related protein [*Oryza sativa*]	2.16	**2.43**	**3.51**		Hl697
ES437672	5,00E-08	Kunitz inhibitor ST1-like [*Medicago truncatula*]	5.39	**2.28**	**2.95**		Hl2037
ES437684	2,00E-65	thioredoxin-dependent peroxidase [*Nelumbo nucifera*]	1.62	**2.23**	1.44		Hl3152
ES437732	1,00E-28	cystatin-like protein [Citrus × paradisi]	1.63	**1.91**	NA	-1.6	Hl285
ES437734	1,00E-11	metal ion binding [*Arabidopsis thaliana*]	-1.5		-1.77	**3.84**	Hl3490
ES437777	4,00E-93	Platanus × acerifolia putative zinc-binding protein	1.88	**2.11**	**2.24**		Hl481
ES437738	1,00E-24	hypothetical protein OsI_022889 [Oryza sativa], AN1-like Zinc finger		**1.98**	**2.27**		Hlct1522
ES437774	7,00E-28	S locus F-box protein with the low allelic sequence polymorphism 2-Sf [*Prunus mume*]		**-7.04**	**-5.52**	**5.47**	Hl4049
ES437770	3,00E-98	acireductone dioxygenase-like protein [*Brassica juncea*]	1.56	**2.23**	1.93		Hl3444
ES437691	2,00E-31	auxin-repressed protein-like protein ARP1 [*Manihot esculenta*]	**-10.78**	-1.93	-1.49	**2.23**	Hlct188
ES437694	2,00E-25	putative auxin-repressed protein [*Prunus armeniaca*]	**-6.17**	**-4.13**	**-2.35**	**2.08**	Hl1264
ES437742	1,00E-65	Mannose/glucose-specific lectin	NA	**2.25**	1.81	**-2.96**	Hl1960
ES437746	2,00E-08	pore-forming toxin-like protein Hfr-2 [*Triticum aestivum*]	**-5.82**	**-4.68**	**-3.22**		Hlct272
ES437782	4,00E-22	carbohydrate binding [*Arabidopsis thaliana*]	**-6.94**	**-3.46**	-1.57		Hl2175
ES437726	1,00E-15	*Triticum aestivum *acidic ribosomal protein			**-2.07**	**2.3**	Hl3792
ES437790	8E-17	Similar to threonine endopeptidase [*Arabidopsis thaliana*]	5.27	**7.32**	**5.53**	**2.51**	Hl3859
ES437784	7,00E-28	unknown protein [*Arabidopsis thaliana*]	1.8	**2.16**	**2.87**		Hl3471
ES437776	4,00E-50	unknown protein [*Arabidopsis thaliana*]	-2.28	**-2.1**	**-2.32**	**2.08**	Hl2053
ES437787	4,00E-51	unknown protein [*Arabidopsis thaliana*]	1.58	**-3.72**	**-2.41**	**2.82**	Hl3322

		***No identity***					

ES437699				**3.19**	**2.99**	1.83	Hl3362
ES437682			**12.98**	**22.04**	**9.41**		Hlct85
ES437795					-1.65	**-3.23**	Hlct367
ES437791			-1.45	-1.6	-1.84	**2.45**	Hl3793
ES437785				**-2.52**	**-2.27**	**2.02**	Hl2561
ES437735			-1.65			**2.01**	Hl1860
ES437731			**7.52**	**11.15**	**5**		Hlct397
ES437686			**5.78**	**12.48**	**7.98**	**2.59**	Hlct182
ES437728					**2.19**		Hl3941
ES437725			1.76	1.81	**2.15**		Hl1453
ES437714			-2.05	**-2.15**	**-2.15**	**2.94**	Hl2078
ES437671			-2.61	**-2.13**	**-2.46**		Hl393
ES437743			1.58	**1.96**	1.84		Hl109

A total of 133 differentially expressed unigenes during different phases of nodule formation were sorted into groups according to their putative physiological role estimated according to TAIR. Clones obtained by a reverse transcriptase–PCR based cloning approach appear in italic. Negative and positive values indicate down- and up-regulation respectively. Values presented in bold format represent those meeting the criteria of a FDR < 0.05 and a fold change ≥ 1.87 or ≤ -1.87. Values without bold format represent a fold change ≥ 1.41 or ≤ -1.41. Note that for the time point T24h versus T0 there are less values in bold format since only two replicates were carried out. NA stands for not available data due to their removal from analysis before normalization.

Some of the 133 contigs may contain paralogs; therefore the contig groups are thought to provide a conservative estimate of the number of genes, i.e the minimum number of genes sequenced. The largest contigs included sequences similar to genes coding for photosystem II type I chlorophyll a/b-binding proteins (64 clones), chlorophyll a/b-binding proteins of light harvesting complex (13 clones), glyceraldehyde-3-phosphate dehydrogenase C subunit (10 clones), auxin-repressed protein-like protein ARP1 (9 clones), oxygen evolving complex 33 kDa photosystem II protein (9 clones), light inducible tissue-specific ST-LS1 gene (8 clones), AAA ATPase, central region, Homeodomain-like (8 clones), major allergen Pru p1 (8 clones), small subunit of ribulose-1,5-bisphosphate carboxylase/oxygenase (7 clones), pore-forming toxin-like protein (6 clones), extensins (6 clones), plastidic aldolase-like protein (4 clones). In addition, three contigs displaying similarity to unknown proteins or no identity contained 13, 8 and 7 clones. All the other contigs included sequences isolated once (96 singletons), twice or three times. The data discussed in this publication have been deposited in NCBI's Gene Expression Omnibus [22] and are accessible through GEO accession number GSE12339 . To identify genes with statistically significant expression changes we used the RP test statistics (see "Methods"). This fully non-parametric test does not require an estimate of the gene expression-specific measurement variation and is therefore more stable with regard to experimental noise and small data sets with low number of replicates [23]. Estimation of false positives (pfp, see "Methods" and Additional file 2) provided a convenient way to determine how likely it is to observe each RP value calculated in/from replicated experiments.

The largest fold changes among all of the unigenes was 22.04 fold up-regulation of a clone with no identity retrieved (Hlct85), and 18.13 fold down-regulation of chlorophyll a/b binding protein (Hl240). Allene oxide synthase and allene oxide cyclase were not differentially expressed at the time points studied, confirming previous expression data [8].

Database analyses revealed that many previously reported plant somatic embryogenesis-related genes were identified, including those encoding chitinases and glucanases [24,25], lipid-transfer proteins [26], glutathione S-transferase [27], tubulin and histone-coding genes [28,29], calmodulin [30], heat-shock proteins [31], *S*-adenosyl-Met synthetase [1], zinc finger-like protein, metallothionein-like protein, senescence-associated protein and epoxide hydrolase [2], which confirmed the comprehensiveness of our cDNA library.

**Figure 2:**
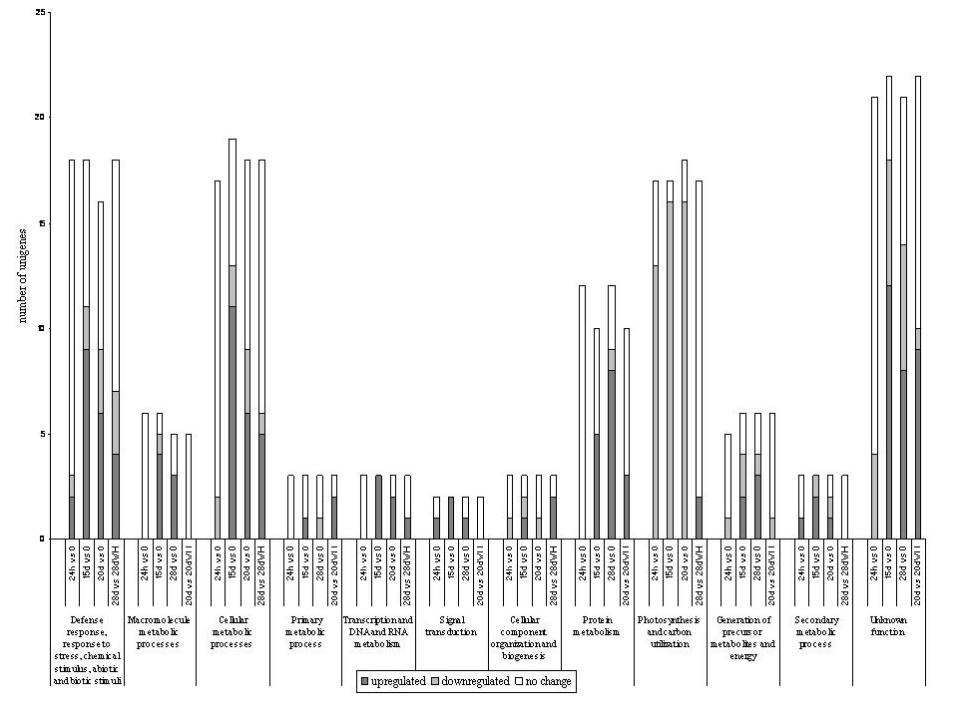
**Distribution of differentially expressed unigenes among several functional classes**. Functional classes were identified using the Arabidopsis protein classification defined by TAIR.

When morphogenic stages (T15d and T28d) were compared with T0 there is increased transcription of genes coding for proteins responsible for defense response, metabolic processes such as those involved in cell wall modification, transcription and DNA and RNA metabolism, signal transduction, cellular metabolic processes such as glycolysis, sugar metabolism, and related to S-adenosyl-L-methionine cycle, protein metabolism and secondary metabolite synthesis (Table 1; Figure 2). Many of these unigenes seem to be up-regulated already after 24 h of culture (Table 1). The most prominent group of transcripts up-regulated during culture in medium with hormones (T15d and T28d) was related to cellular metabolic processes, and with unknown function (12 unigenes in each class). On the other hand, the genes that appear down-regulated at these morphogenic stages are mostly genes sharing functional annotations related to light reactions of photosynthesis and Calvin cycle (18 unigenes).

Unigenes involved in stress responses, related to S-adenosyl-L-methionine cycle, signal transduction and secondary metabolism were up-regulated in events leading to prenodule formation (T15d) followed by a decrease in their expression during nodule formation (T28d). On the other hand, many genes were more expressed in organogenic nodules (T28d) than in T24h or T15d, for instance: early flowering 3 (Hl3446), endotransglycosylase/hydrolase precursor XTH-30 (Hl3465), Avr9/Cf-9 induced kinase 1 (Hl3459). However, most of these genes do not show any difference in expression when compared to the T28dWH; thus, their role in organogenic nodule formation cannot be ascertain and may rather be related to a response of explants to *in vitro *conditions. Remarkably, one gene coding for a lipid transfer protein (Hl2898) was up-regulated in organogenic nodules (T28d) when compared both to T0 and to non-morphogenic conditions (T28dWH), suggesting that this gene can be regarded as a marker of organogenic nodule formation.

When samples corresponding to T28d were compared to T28dWH a tendency of variation within the functional classes could not be established. The differences between organogenic and non-organogenic structures seem to rely on variation of specific genes representing several functional classes. We could observe down-regulation of genes coding for proteins related to stress response such as Senescence-Associated Gene 21, a putative gamma-thionin, and a thiazole biosynthetic enzyme (Table 1), as well as up-regulation of peroxidases and mandelonitrile lyase. This last one has not been previously assigned to morphogenic processes. Genes coding for kinases namely a MAP kinase-like protein, and a Protein kinase (Type I EGF), and related to protein metabolism and transport were up-regulated indicating that morphogenesis also involves changes in translational and post-translational modifications.

#### Verification of Microarray data by Quantitative RT-PCR

**Figure 3:**
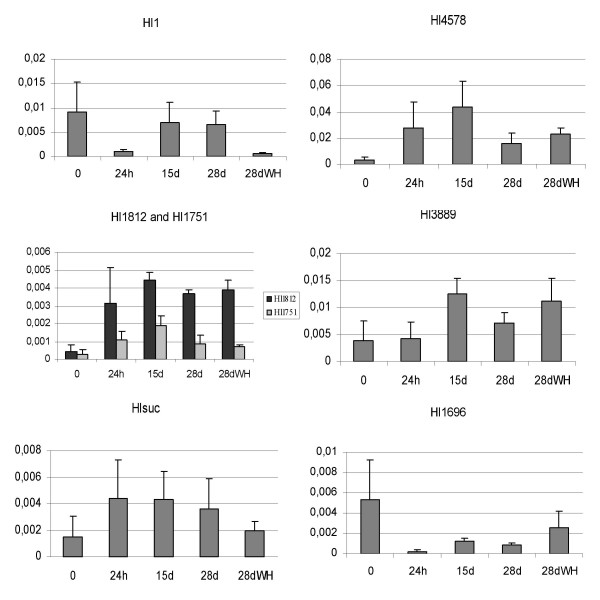
**Quantitative RT-PCR analysis**. Complementary cDNAs were used encoding a metallothionein (Hl3889, Hlct1310), sucrose synthase (Hlsuc), glycolate oxidase (Hl1696), 3-deoxy-D-arabino-heptulosonate 7-phosphate synthase (Hl1812), cinnamic acid hydroxylase (Hl1751) and peroxidases (Hl1 and Hl4578). Hl1812 and Hl1751 are both involved in secondary metabolism and were grouped together. Values are the mean of three-four experiments; bars represent SE. Graphs are plotted against relative cDNA concentration (Y axis) assessed by plasmid.

In order to validate data obtained from the microarray studies genes with different expression profiles and/or belonging to different functional classes were independently quantified by quantitative RT-PCR. They include cDNAs encoding a metallothionein (Hl3889), sucrose synthase (Hlsuc), glycolate oxidase (Hl1696), 3-deoxy-D-arabino-heptulosonate 7-phosphate synthase (Hl1812), cinnamic acid hydroxylase (Hl1751) and peroxidases (Hl1 and Hl4578). The quantitative RT-PCR results showed similar expression patterns as obtained for microarrays (Figure 3). Two cDNAs coding for peroxidases showed indeed different expression profiles. Complementary DNAs coding for enzymes involved in the synthesis of secondary metabolites (Hl1812 and Hl1751) showed similar expression profiles. In all cases, we obtained a strong correlation between the results obtained in microarray experiments and in quantitative real-time RT-PCR. This gives support to the predictions made based on the microarray experiments and demonstrates the reliability and sensitivity of the microarray slides developed.

### Metabolic profiling

#### Metabolic elucidation using one and two dimensional NMR spectroscopy

**Figure 4:**
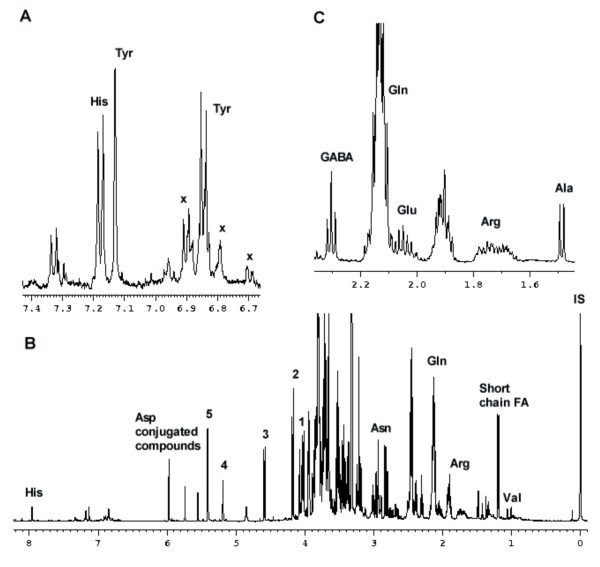
**^1^H NMR analysis in a sample corresponding to prenodular stage (T15d)**. **A **^1^H NMR spectra corresponding to a T15d sample in the range of *δ *6.66 to *δ *7.43. X, dihydrophenylpropanoids. **B **^1^H NMR spectra corresponding to a T15d sample in the range of δ 0–8.2. 1, *myo*-inositol; 2, H-1 of Fru in Suc; 3 H-1 of β-Glc; 4, H-1 of α-Glc; 5, H-1 of Suc; IS, internal standard (TSP); FA, fatty acid.**C **Expansion in the range of *δ *1.43 to *δ *2.38. Peak of Gln was partially deleted.

Variations in mRNAs levels are likely to be involved in the physical and metabolic changes that occur during morphogenesis. ^1^H NMR together with 2D J-resolved and COSY (correlated spectroscopy) techniques are a reliable methodology for recognition of a broad metabolome, detecting compounds such as amino acids, carbohydrates, organic acids and phenolic compounds. The two-dimensional techniques were applied to overcome the congestion of ^1^H NMR spectra and improve their resolution [17]. Figure 4 shows a ^1^H NMR spectrum of the metabolome of a hop sample corresponding to 15 days of culture. Signals at *δ *5.40 (d, *J *= 3.5 Hz), *δ *5.18 (d, *J *= 3.5 Hz), *δ *4.58 (d, *J *= 7.5 Hz) and *δ *4.17 (d, *J *= 8.5 Hz) were assigned to be anomeric protons of glucose moiety of sucrose, α-glucose, β-glucose and fructofuran moiety of sucrose, respectively (Figure 4B). Amino acids were identified at *δ *7.85 (d, *J *= 1.0 Hz) and *δ *7.09 (brs) as histidine, at *δ *7.18 (d, *J *= 8.8 Hz) and *δ *6.86 (d, *J *= 8.8 Hz) as tyrosine, at *δ *3.94 (m), *δ *2.96 (dd, *J *= 3.5 Hz, *J *= 17.0 Hz) and *δ *2.82 (m) as asparagine, at *δ *2.46 (m) and *δ *2.14 (m) as glutamine, at *δ *2.39 (m) and *δ *2.04 (m) as glutamate, at *δ *1.92 (m) and *δ *1.72 (m) as arginine, at *δ *1.48 (d, *J *= 7.5 Hz) as alanine, at *δ *1.34 (d, *J *= 6.5 Hz) as threonine and at *δ *1.06 (d, *J *= 7.0 Hz) and at *δ *1.01 (d, *J *= 7.0 Hz) as valine. In addition to these compounds, adenine, *myo*-inositol (inositol), choline, *γ*-aminobutyric acid (GABA), a short chain fatty acid and trace amounts of α-linolenic acid were identified at *δ *8.19 (s), *δ *4.03 (t, *J *= 8.5 Hz), *δ *3.22 (s), *δ *2.31 (t, *J *= 7.5 Hz), *δ *1.20 (d, *J *= 7.0 Hz) and *δ *0.95 (t, J = 6.5 Hz), respectively (Figures 4A, B, C).

In the aromatic region (*δ *5.7–9.0) phenolic signals were detected at low levels but mostly in prenodules (T15d) (Figure 4A). Some of these peaks correlated with tyrosine region in HMBC spectra (heteronuclear multiple bond correlation) and were assigned as dihydrophenylpropanoids (see Additional file 3).

**Figure 5:**
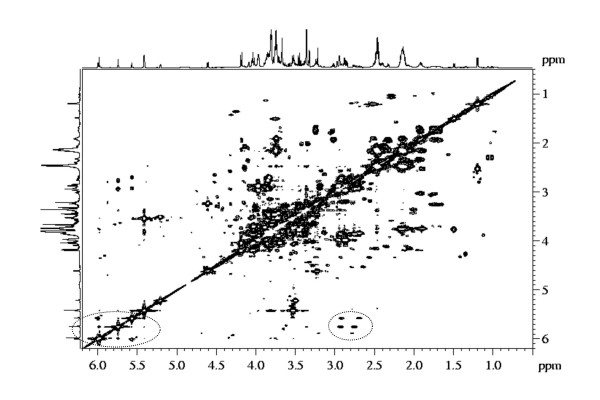
**COSY analysis in a sample corresponding to prenodular stage (T15d)**. Spectrum corresponds to a T15d sample in the range of *δ *0.5 to *δ *6.2 ppm. Circles highlight correlations of signal at *δ *5.55 with *δ *5.74 and *δ *5.97; and also with signals in aspartate region.

In order to identify peaks at *δ *5.55 (d, *J *= 1.5 Hz), *δ *5.74 (s), *δ *5.97 (d, *J *= 2.0 Hz) two dimensional techniques including J-resolved, COSY and HMBC were used. In COSY spectrum the signals at *δ *5.55 correlated with *δ *5.74 and *δ *5.97. Also, it correlates with the signals in aspartate region (Figure 5). HMBC showed correlation of peaks at *δ *5.74 and at *δ *5.97 with *δ *138 and *δ *172.5. Moreover, HMBC showed correlation of peaks at *δ *5.55 and at *δ *5.97 with *δ *141 and *δ *174.5 (see Additional file 3). Taking into account all the data these peaks were identified as corresponding to aspartate-conjugated metabolites.

**Figure 6:**
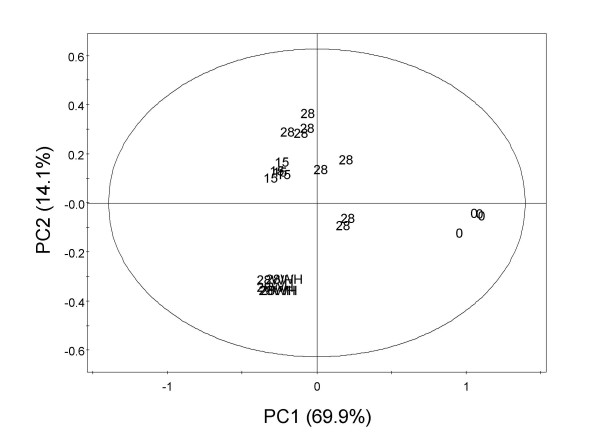
**Score scatter plot discriminating morphogenic stages by using metabolic profiling coupled to principal component analysis**. Spectral intensities were scaled to total intensity and reduced to integrated regions of equal width (0.04 ppm). The ellipse represents the Hotelling T2 with 95% confidence in score plots.

**Figure 7:**
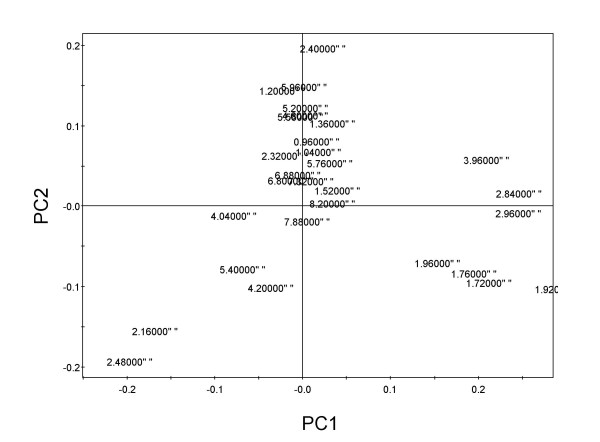
**Loading scatter plot for principal component analysis**. A Kruskal-Wallis test was performed using spectral intensities scaled to total intensity and reduced to integrated regions of equal width (0.04 ppm). The values for the loading scatter plot were selected when significantly different at a *p *value lower then 0,005.

Principal component analysis is an unsupervised clustering method requiring no knowledge of the data set and acts to reduce the dimensionality of multivariate data while preserving most of the variance within it [32]. Transcriptomic analysis showed that T15d and T28d are developmental stages with induction and repression of transcription of many common genes. Regarding metabolomic analyses, T15d and T28d also share similar metabolic profiles as demonstrated from the score scatter plot (Figure 6). The biological variation obtained for T28d, both for transcriptomics and metabolomics, can be due to lack of synchronization in the development of nodules [4]. Morphogenic stages (T15d and T28d) are characterized by higher PC2 values then control explants (T0d and T28dWH). PC1 accounts for 69.9% of variation whereas PC2 accounts for 14.1%. Moreover, T0 showed higher PC1 values then the other samples (Figure 6). In order to identify which metabolites were present in significantly different amounts among all time points we performed a Kruskal-Wallis test (see Material and Methods, see Additional file 4) using spectral intensities at different chemical shifts (*δ *= 0.4–10.0) and reduced to integrated regions of equal width (0.04 ppm). The values which were significantly different at a *p *value lower then 0.005 using this non-parametric test, and were previously identified by ^1^H NMR spectra and two dimensional techniques were selected for the loading scatter plot (Figure 7). The compounds responsible for more variance among the four time points were glutamine, sucrose and inositol for lower PC2 and PC1 values (T28dWH); asparagine and arginine for higher PC1 and lower PC2 (T0); glutamate, glucose, threonine, aspartate conjugated compounds, a short chain fatty acid and α-linolenic acid for higher PC2 values and lower PC1 values.

In order to discriminate between T15d and T28d samples and between T28d and T28dWH samples a Wilcoxon Rank sum test was performed using spectral intensities at different chemical shifts (see Additional file 4). Together with analysis of ^1^H NMR spectra it was concluded that T15d samples accumulate more tyrosine, dihydrophenylpropanoids, aspartate conjugated compounds, a short chain fatty acid and sucrose than T28d samples. In contrast, T28d samples accumulate more α-linolenic acid and more amino acids such as asparagine, glutamate, alanine and valine than T15d and T28dWH samples.

Visual inspection of spectra showed accumulation of choline in T15d and T28d samples. However, the intensity of this signal could not be analyzed statistically due to congestion of this signal at *δ *3.22 with a glucose signal (*δ *3.24).

#### Quantification of thiols by HPLC

**Figure 8:**
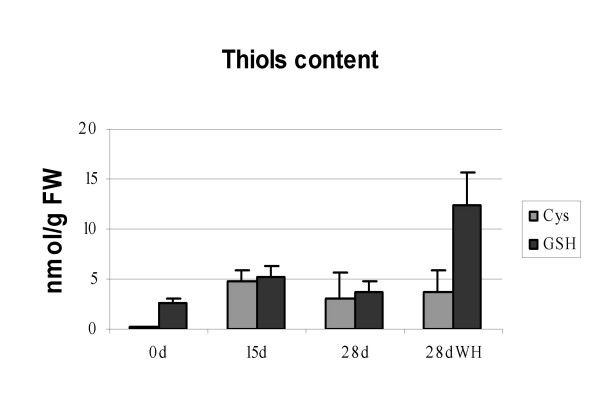
**Quantification of cysteine and glutathione during organogenic nodule culture**. Values are the mean of three-four independent experiments; bars represent SE.

Transcriptional and metabolome profiling indicated strong changes in redox status of tissues. Thiols are often mentioned in the context of oxidative stress response. As shown in Figure 8, high amounts of cysteine and glutathione were detected in explants cultured *in vitro*. The increase in cysteine between T15d and T0 was about 18.69 fold whereas the increase in glutathione was about 2 fold. Between T15d and T28d there were a 1.52 and 1.42 fold decrease in cysteine and glutathione respectively. In non-morphogenic samples (T28dWH) the values of glutathione were 12.41 nmol g^-1 ^fresh weight. Interestingly, samples cultured on medium without hormones showed 3.42 fold more glutathione then T28d samples. On the other hand, cysteine values were similar (3.76 and 3.10 nmol g^-1 ^fresh weight, for non-morphogenic samples and organogenic nodules, respectively). Glutathione biosynthesis is a key component in the network of plant stress responses that counteract oxidative damage and maintain intracellular redox environment. The higher content of thiols such as glutathione on T28dWH samples might be an indicator of the highly oxidizing environment in non-morphogenic tissue.

## Discussion

In this work, an integrative transcriptome/metabolome analysis of organogenic nodule formation was conducted in cultured hop tissues. Analyses of the transcriptome and metabolome suggested that cells respond to *in vitro *culture by activating mechanisms of defense, changing from a partially autotrophic to a heterotrophic metabolism due to up-regulation of genes involved in macromolecule and cellular metabolic processes such as glycolysis and sucrose degradation as well as the down-regulation of genes for coding for photosynthesis. For discussion purposes, we grouped these genes into functional classes and provide an integrative model of putative interactions.

### Defense/stress response, response to chemical stimulus and response to abiotic and biotic stimuli

Organized development in cultured tissues is promoted by stress treatments [33]. Wounding and osmotic stresses due to internodes inoculation in culture medium with sucrose may play a role in organogenic nodule induction. Genes coding for peroxidases (Hl4206, Hl4578), allergens (Hl1695, Hlct313), chitinase (Hlct1044), and glutathione S-transferase (Hl1525) are generally involved in stress response [34] and seem to be more expressed up to prenodular stages.

Interestingly, other genes related to stress response such as one coding for a putative gamma thionin (Hl1925) and a senescence associated gene 21 (Hl4571) were more expressed in T28dWH samples when compared to T28d. This expression pattern suggests that these non-morphogenic tissues are extremely oxidized. Accordingly, the levels of *myo*-inositol, which plays a role in plant defence to stress [35] increased in T28dWH comparing to T28d.

Glutathione-S-transferase (Hl1525) was induced throughout development which was in agreement with the also detected increased glutathione levels. A large accumulation of glutamine, a metabolite involved in glutathione synthesis was detected in T28dWH samples. This major antioxidant pool (glutathione) plays a role in plant defense and promotes somatic embryogenesis in spruce [36]. So it is not surprising that increased glutathione levels were detected in T15d, T28d and T28dWH samples.

Cell wall peroxidases are involved in morphogenesis [37]. A cDNA coding for a cationic peroxidase (Hlct1) was up-regulated in organogenic nodules comparing to T28dWH suggesting that its gene products are related to morphogenesis and not solely to stress caused by culture conditions.

In somatic embryos, genes coding for Hsps are differentially expressed [38]. In tomato, a plastid-localized Hsp (pTOM111) increased several fold in ripening fruit and in response to heat stress; it has been implicated on the reorganization of thylacoid membranes during the transition from chloroplast to carotenoid-accumulating chromoplasts [39]. Induction of an Hsp90-like mRNA (Hlct1560) in prenodules and in organogenic nodules may be related to the transition of chloroplast to amyloplast and amyloplast to chloroplast. Indirect support for this hypothesis is the observation that Hsps are induced in explants cultured without hormones where amyloplasts are also formed [4].

A transcript coding for a mandelonitrile lyase (Hl2222) was found to be up-regulated in organogenic nodules comparing to T28dWH. Mandelonitrile lyase participates in the hydrolysis of cyanogenic glycosides which function in nitrogen storage for germination and plantlet development, and pathogen and herbivory defense [40] but has not been previously assigned to *in vitro *morphogenic processes. Interestingly, it is also worth noting that valine, a precursor of cyanogenic glucosides, is increased in T28d samples (see Additional file 4).

### Macromolecule, cellular and primary metabolic processes

Cell wall modification enzymes may be on the basis for the changes in cell proliferation and vascular tissue formation of *in vitro *cultured tissues [24,41]. During prenodular and nodular stages it was detected up-regulation of genes related to cell wall-modifications such those coding for a glucanase (Hlgluc5), a pectinesterase (Hl52), an extensin (Hlct117) and a xyloglucan endotransglycosylase/hydrolase precursor (Hl3465). Interestingly, the expression of an endoglucanase inhibitor transcript (Hl3829) was found to change. The involvement of this gene in morphogenesis has not been previously reported. The down-regulation of an endoglucanase inhibitor transcript all through nodule induction and formation and its up-regulation in morphogenic tissues (T28d) (comparing to T28dWH) indicates a tight post-transcriptional regulation of cell-wall modifying enzymes.

Transcription induction of β-1,3-glucanases at prenodular and nodular stages might be associated to the degradation of callose necessary for the nodule formation and subsequent plant regeneration [42]. Indeed, β-1,3-glucanases have been assigned to callose degradation during somatic embryogenesis [24,43].

A striking feature of a gene coding for an extensin is its several fold increase from 24h up to prenodular formation, probably related to cell wall plasticity.

Additionally, a wall-associated protein kinase Type I EGF transcription (Hl4559) was increasingly repressed during organogenic nodules' formation but shows higher transcriptional level in T28d than in T28dWH samples. Up to our knowledge this gene has not previously been related to *in vitro *plant morphogenesis. By interacting with cell wall pectins [44], wall associated kinases may play a role in cell elongation and cell differentiation during morphogenesis.

Cell wall synthesis, starch production, and respiration require hexoses. The decrease in photosynthetic activity in plant cells cultured *in vitro *makes the addition of exogenous sugar, in particular sucrose, to the culture medium an absolute necessity for nearly all tissues. The carbohydrate pool (glucose and sucrose) was clearly increased in *in vitro *cultured samples (T15d, T28d, T28dWH). Accordingly, sucrose synthase gene was up-regulated at prenodular stage (T15d) accounting for the importance of sucrose degradation. Sucrose is an inducer of organogenic nodule formation in hop whereas glucose is inefficient [4], feature also documented for embryo development [11]. Sugars play a central role in the control of plant metabolism, growth, and development and have interactions that integrate light, stress, and hormone signaling [45,46]. They regulate the expression of lipoxygenase genes, pathogenesis-related (*PR*) genes, and other stress-inducible genes [45] which were shown to be differentially expressed in this work.

Sucrose uptake and breakdown can originate the observed glucose accumulation in T15d and T28d samples. This accumulation is unlikely derived from *de novo *photosynthesis since transcripts levels of genes related to photosynthesis were decreased. Though sucrose synthase gene (Hlsuc) was not differentially expressed in T28d versus T28dWH samples, the latter showed more sucrose. In fact, sucrose reached its higher levels in non-morphogenic samples suggesting that is not being mobilized upon uptake from the medium leading to low levels of glucose which may be impairing growth and morphogenesis, both high energy-requiring processes. Interestingly, a gene coding for a mannose/glucose specific lectin (Hl1960), which is a carbohydrate-binding protein was up-regulated in T28dWH comparing to T28d.

The importance of sugars interconversion in morphogenesis was further suggested by the up-regulation of transcripts for a pfkB-type carbohydrate kinase (Hl3980) and for a dTDP-glucose 4-6-dehydratase (Hl4620).

During prenodules formation there was induction of cytosolic glyceraldehyde-3-phosphate dehydrogenase (GAPDH, Hlct23) and fructose-1,6-biphosphate aldolase (Hl3973) isoenzymes. In opposition, the correspondent plastidic isoenzymes were down-regulated (Hl263 and Hlct77 respectively). This is not surprising since chloroplasts are converted to amyloplasts at this stage [4]. Cytoplasmic fructose-biphosphate aldolase and GAPDH are glycolytic enzymes. The glycolysis pathway can provide carbon skeletons to the TCA cycle, lipid metabolism and phenylpropanoid-flavonoid pathway. Metabolic profiling showed that signal assigned to be dihydrophenylpropanoids increased at prenodular stage which is in accordance to an induction of glycolytic enzymes in T15d samples. Dihydrophenylpropanoids (e.g. dihydrocinnamic acids) are involved in the biosynthesis of phenylphenalenones which make part of the defense system of certain plant species [47].

Besides changes in organic acid metabolism, changes in lipid metabolism were also noticed during development of nodules. Lipoxygenases have been related to somatic embryos formation [1]. A *de novo *synthesis of three LOX isoenzymes was observed during organogenic nodule formation in hop [6]. The two ESTs coding for LOXs that were found in this work could correspond to enzymes located in different compartments and/or have different metabolic activities. Hllox6 was up-regulated until the stage of prenodule formation. The expression levels of another gene coding for a lipoxygenase (Hl2195) and a gene coding for glycerophosphodiesterase (Hl3510) are higher in T28d than in T28dWH samples (Table 1). It is also noteworthy that T28d samples have increased levels of choline, a short chain fatty acid and α-linolenic acid comparing to T28dWH, which stresses the role of lipid metabolism in morphogenesis as previously shown [42].

Lipid transfer proteins are expressed during somatic embryogenesis and are possibly involved in the transport of cutin monomers [26]. A lipid transfer protein precursor encoding gene (Hl2898) was up-regulated in T28d when compared to both T0 and T28dWH, strongly indicating that this gene constitutes a marker of organogenic nodule formation, likely related to the previously reported deposition of cutin specifically in morphogenic regions of nodular structures that will give rise to plantlets [42]. A gene coding for epoxide hydrolase (Hl1072), involved in the β-oxidation of epoxy fatty acids, important constituents of the cutin layer also appeared up-regulated [48].

Phytohormones are widely described as inducers of morphogenesis. S-adenosyl-L methionine (SAM) provides methyl groups in many biological methylations and acts as a precursor in the biosynthesis of the polyamines spermidine and spermine, and of the gaseous hormone ethylene [49]. Here it was a found a peak in transcription of genes coding for SAM synthetase (Hlct214), SAM descarboxilase (Hl3196) and a cobalamine-independent methionine synthase (Hl4592) during prenodular stages. This suggests increased SAM synthesis most probably related to the huge increase in polyamines previously detected in prenodules and nodules [9]. Polyamines have been implicated in plant cell proliferation and differentiation, morphogenesis, embryogenesis, and also in senescence and stress responses [50,51]. Putrescine, a polyamine synthesized upon wounding can be use in the synthesis of GABA via putrescine catabolism [50]. As in other stress situations, the non-protein amino acid GABA accumulated throughout hop culture in particular during nodule formation. An opposite trend was observed for nitrogen-rich amino acids such as asparagine and arginine which presented higher levels in T0. The low levels of arginine in morphogenic samples can be due to increased polyamine synthesis through arginine decarboxilase activity [9].

Signaling of wounding, pathogens, plant hormones, and cell cycle cues is transduced by Mitogen-activated protein kinases (MAPKs) [52]. In addition to the previously described hop Extracellular signal-regulated kinase 1 and 2 (ERK1/2) [7], here we found induction of a gene coding for a MAPK (Hl4516) in organogenic nodules, when compared to T28dWH, suggesting that also this MAPK may be involved in signalling processes that give rise to nodule formation.

The clone Hl3157 coding for Plant lipid transfer protein/Par allergen presented a peak of expression during prenodular stages, which may suggest that it constitutes a marker of morphogenic competence. Interestingly, in TAIR this gene also presented a significant similarity to a putative receptor serine/threonine kinase (see Additional file 1). A somatic embryogenesis receptor kinase (SERK) is involved in the acquisition of embryogenic competence in plant cells [53].

### Transcription and DNA and RNA metabolism, signal transduction, protein metabolism and cellular component organization/biogenesis

Histones modification may affect the expression of patterning genes during morphogenesis [29]. Homologs of histones (Hl2418 and Hlct2847) found during organogenic nodule development in hop may be involved in chromatin remodelling and cell proliferation processes. Histone H4 gene was up-regulated in organogenic nodules comparing to T28dWH. In addition, two transcripts encoding ribosomal proteins (Hl3792 and Hl3805) and another transcript coding for a ribosome inactivating protein (Hl3627) were up-regulated when comparing these two samples, which indicate a tight control of proteins synthesis during morphogenesis. Cytoskeleton and its regulators are essential for proper cell morphogenesis [54]. Microtubule formation during somatic embryogenesis in carrot is coordinated with concomitant changes in tubulin-gene transcription [28]. In this study, we observed a 7.36 fold increase of α-tubulin (Hl2210) in organogenic nodules compared to T28dWH. It seems that microtubule arrays do not form by the reorganization of pre-existing microtubules but that new microtubules assembly occurs, suggesting a reinforcement of the microtubular cytoskeleton in morphogenesis.

In our transcription profiling genes coding for Rac/Rop GTPases presented a peak of expression during prenodular stages (Hl1537 and Hl1506). In plants, Rac/Rop GTPases play important roles in defense response, establishment of cell polarity, and hormone signalling [55]. During prenodule formation, other genes related to signals transduction, such as calmodulin (Hl2815), a calcium sensor protein, were up-regulated. The expression of the three wheat calcium-regulated genes support a specific role for Ca^2+ ^in somatic embryogenesis [56]. We found significant induction of calmodulin in T15d samples suggesting that calmodulin may participate in the determination of prenodular cells to develop into nodules. An important role of calcium on organogenic nodule formation in hop has been previously suggested [57].

### Photosynthesis, carbon utilization and generation of precursor metabolites and energy

Oxygen is limiting in developing embryos due to the confined environment of *in vitro *culture. Thus, photosynthesis in embryos, even if operating at a low rate, is important for oxygen supply. Here we found that several genes coding for proteins putatively related to photosynthesis were down-regulated immediately after 24 h of culture, in prenodules and, to less extent, in nodular explants due to a re-greening process of these latter [4]. Among the proteins identified during somatic embryogenesis, Rubisco small chain proteins gradually decrease [58]. The down-regulation of housekeeping proteins such as Rubisco may be related to jasmonic acid levels [[59], reviewed by [60]], which peaked in hop internodes cultured for 24 h [8]. The decrease of photosynthesis transcripts may indicate an adjustment of photosynthetic rates, often associated with a specific role in protection against oxidative stress. However, the down-regulation of genes involved in photosynthesis seems to be related to a response to *in vitro *culture and not specifically involved in morphogenesis.

During *in vitro *culture O_2 _concentration is low, thus it is not surprising to find down-regulation of a gene coding for a photorespiration enzyme, glycolate oxidase/oxidoreductase (Hl1696).

Growing embryos are predominantly heterotrophic, producing ATP via glycolisis and respiration [61]. The induction of a gene coding for an F1-ATP synthase delta subunit (Hl1512) supports an increased ATP pool. The increase in ATP synthase transcription levels in prenodules and nodules may be related to an increase in ATP synthesis and its transport to amyloplasts where starch is being accumulated. The same can take place in T28dWH samples eventually at a lower level since these samples accumulate less starch [4].

Interestingly, a gene coding for a putative steroid binding protein (Hl1863) was down-regulated in nodules comparing to T28dWH samples. Moreover, it did not present significant differences in expression throughout culture. The increase in transcription of this gene may be regarded as marker of non-morphogenic samples.

### Secondary metabolic process

In this study, an increase in secondary metabolites in samples with increased carbohydrate pool was detected. A similar situation has been described for the Arabidopis *pho3 *mutant, which accumulates sucrose and other carbohydrates to high levels [46].

During prenodular stages, it was found a peak in transcription of genes coding for a cinnamate 4-hydroxylase (Hlct225), a putative flavonol synthase (Hl3668) and a phenylalanine ammonia lyase (Hlpal); enzymes involved in phenylpropanoids and flavonoids synthesis. Accompanying the transcriptional induction of phenylalanine ammonia lyase a gene coding for a 3-Deoxy-D-arabino-heptulosonate-7-phosphate synthase (DAHPS, Hl1812) was found up-regulated in early stages of nodule culture. One of the possible end-product of this pathway is the synthesis of chorismate which in turn leads to tryptophan, phenylalanine and tyrosine. This latter aromatic amino acid also accumulated during prenodule formation (T15d).

Moreover, phenolic compounds such as dihydrophenylpropanoids were detected mostly in prenodules (T15d). Phenolic compounds, in particular, flavonoids have been referred to be involved in the initiation of root nodules in legumes through their action as auxin transport inhibitors [62]. Though these nodules presented different morphological and metabolic features from organogenic nodules, it can be speculated that phenolic compounds are involved in establishment of both nodular processes eventually by regulating auxin transport.

### Unknown function/No identity

It is not possible to establish a function based on annotation, and, in some cases, even to find homologues for a number of genes induced during organogenic nodule formation. However, searches in the literature together with previous research carried out in hop organogenic nodules could bring insights concerning the possible function of some of these differentially expressed genes. Examples are two classes of auxin regulated transcripts identified in this study: auxin-repressed protein (ARP) coding gene (Hlct188, Hl1264) and Aux/IAA early auxin-response gene (Hl4491). The importance of auxin for acquisition of morphogenic competence in hop cultures was demonstrated by the fact that auxin absence in induction medium would delay or impair nodule formation [4]. Steady-state mRNA levels for one Aux/IAA was shown to decrease with the ongoing of morphogenesis. This may allow for a gradually increasing number of functionally active auxin-response factors proteins and hence a transcriptional activation of auxin-response genes. A variety of ARP proteins are involved in diverse developmental processes [63], indicating that ARP gene expression is low in actively growing tissues. There is down-regulation of two ARP proteins when compared to the control. Nevertheless, this repression seems to be released with the ongoing of this morphogenic process. These results suggest that ARP genes must be down-regulated for early auxin-mediated responses to occur. Strikingly, ARPs and Aux/IAAs were up-regulated when organogenic nodules (T28d) were compared with T28dWH samples. Thus, transcriptional control of auxin signaling and auxin responsive genes seems to underline the differences in morphogenic competence.

Increasing evidence indicates that signal transduction depends on the proteolysis of certain transcriptional regulators. During organogenic nodule formation genes coding for a cystatin-like protein (Hl285), a S locus F-box (Hl4049), and a threonine endopeptidase (Hl3859) are differentially expressed indicating the importance of tight control of protein degradation.

Several ESTs for which no identity was found can be regarded as potential markers of morphogenesis due to the extremely high fold change obtained in prenodules (Hlct85, Hlct397 and Hlct182). The EST corresponding to clone Hlct182 is probably the best candidate among these genes since it is also up-regulated when comparing morphogenic and non-morphogenic samples.

### Network of events leading to organogenic nodule formation in hop

**Figure 9:**
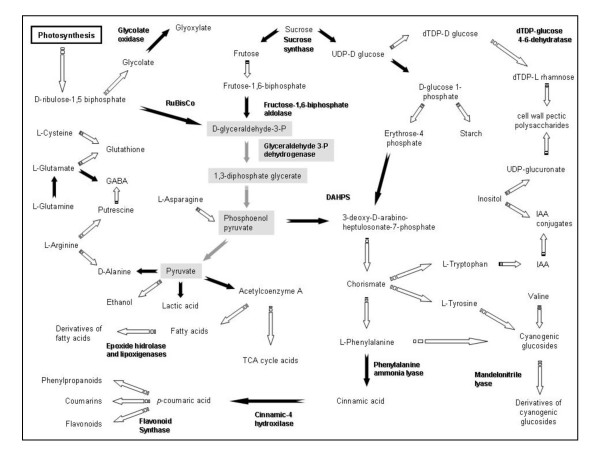
**Metabolic pathways leading to synthesis of metabolites found differentially present in hop organogenic cultures**. Metacyc database was used to elucidate metabolic networks . For simplicity reasons all reactions were shown as unidirectional. Full and dashed arrows represent direct and indirect biochemical reactions respectively. Glycolysis I pathway is highlighted in grey boxes. Genes which were found differentially expressed and could be integrated with metabolomics data are shown. DAHPS, 3-deoxy-D-arabino-heptulosonate-7-phosphate synthase.

Although fundamental developmental processes may be shared among species, there are also remarkable developmental differences, even between species of the same family. This is one reason why effort was put into an integrative genomic and post genomic study of hop nodule cultures (Figure 9).

Our results support the involvement of several stress response-related genes in morphogenesis. The increase in expression profile of some genes such as SAG 21 and in levels of metabolites such as glutamine and glutathione in T28dWH samples suggests a response similar to senescence in those control explants. The fact that T28dWH samples contain more glutathione then T28d samples, supports previous data that increased glutathione biosynthetic capacity in the chloroplast resulted in greatly enhanced oxidative stress [64]. Oxidative stress responses might be triggered in the absence of exogenously supplied hormones, but most probably the mechanisms that ensure the redox balance necessary for the progression of the morphogenesis are lacking. Oxidative stress could enhance an auxin-driven process leading to cell division and to the formation of morphogenic cell clusters. Certain peripheral cells of the morphogenic nodular cluster also enter programmed cell death [6] but oxidative stress must be tightly controlled when considering the whole nodular structure. The study of somatic embryogenesis in soybean revealed that the arrangement of new cells into organized structures might depend in a genetically controlled balance between cell proliferation and cell death [1].

The peripheral senescent cells of nodules may contribute for dilution effects when observing expression of genes and metabolites levels in the growing parts of nodules because nodules were not isolated from the peripheral tissues for sampling. However, these peripheral cells may play an indirect role in morphogenesis by supplying nutrients to the growing nodule. Integrated nutrient management could involve reallocation of nutrients via regulation of transporters, storage of nutrients in energy-rich compounds, and recovery of nutrients from senescent cells to sink organs, the organogenic nodule. Polyamines and glutathione could play a role in the storage strategy of nitrogen and sulphur respectively, together with their involvement in stress response.

Interestingly, several features in organogenic nodule formation could be compared to the mechanisms controlling tumor development in Arabidopsis induced by agrobacteria [65]. Prenodules and organogenic nodules also possess a heterotrophic and anaerobic metabolism. Hop internodal cells removed from parental plants cultured *in vitro *are clearly in a hypoxic environment due to down-regulation of photosynthesis that occurs coincidentally with differentiation of chloroplasts into amylopasts [4]. Under these conditions cells switch to a fermentative energy metabolism. T28dWH samples, however, seem to uptake sucrose from culture medium but not to metabolize it at high rates. Signals derived from increased sugar levels lead to inhibition of genes involved in photosynthesis, Calvin cycle and chlorophyll synthesis, and activation of genes of secondary metabolism [45,46]. At the best of our knowledge, the involvement of genes coding for pfkB-type carbohydrate kinase and for a dTDP-glucose 4-6-dehydratase on *in vitro *plant morphogenesis is being suggested for the first time with this study in hop. This may indicate some differences in carbohydrate metabolism during morphogenesis in different plant species. We believe that inhibition of such pathway by knocking down sucrose synthase, glyceraldehyde-3-phosphate dehydrogenase or carbohydrate kinase may impair organogenic nodule formation even in samples cultured on medium containing growth regulators. In fact, if sucrose is not metabolized, the glycolytic pathway will be down-regulated since *de novo *synthesis of sugars is not possible due to photosynthesis inhibition (Figure 9). This would affect metabolism of lipids, secondary metabolites such as dihydrophenylpropanoids and growth regulators production such as auxins (Figure 9), which were shown to play an important role in organogenic nodule development.

## Conclusion

In this work we were interested in the mechanisms underlying reprogramming of cells through stress and hormone treatments. To the best of our knowledge this study reports for the first time integrated data on both transcriptome and metabolome for *in vitro *morphogenic processes, revealing new features of cells involved in morphogenesis.

Five main pathways seem to be determinant in organogenic nodule formation, namely defense and stress response, sugar and lipid metabolism, secondary metabolism and hormone signaling. It is suggested that cultured tissues respond to *in vitro *conditions by an early activation of defense mechanisms already noticed after 24 h of culture. During prenodule formation (T15d) there is a strong metabolization of sucrose, activation of glycolisis and synthesis of secondary metabolites. Polyamines and auxins seem to be involved in prenodular and nodular formation where intense cell proliferation and differentiation is occurring.

## Methods

### Plant material and culture conditions

The internodes from *Humulus lupulus *(var. Nugget) plants, maintained under *in vitro *conditions, were induced according to the protocol previously described [4]. Internodes were wounded throughout by several incisions using a razor blade (wounding treatment) before inoculation in MS medium [66] with IAA, BAP and sucrose. Material was sampled 4–6 times from independent experiments at the following morphogenic stages: internodes at the time of excision from the parent plant (control); 24 hours upon internodes inoculation; 15 days on culture medium in which several prenodular structures are formed inside the *calluses*; 28 days after culture initiation corresponding to nodule formation. Another control was carried out using internodes cultured for 28 days in medium without growth regulators, which never formed nodules [4].

### RNA extraction and cloning of homologs from hop

Total RNA was isolated essentially as described by Rerie et al. [67] from internodes and from material at various morphogenic stages. To further purify RNA, DNAse treatment was carried out according to suppliers' instructions (Invitrogen, San Diego, CA, USA). Samples were then extracted in phenol/chloroform/isoamylalcohol (75:24:1, v/v/v), precipitated with sodium acetate and ethanol, washed in 70% ethanol and dissolved in water. For hybridizations of microarrays RNA was further purified using RNeasy Plant Mini kit (Quiagen, Valencia, CA, USA).

For cloning of hop glucanase [GenBank:AY795910], chitinase [GenBank:AY849555], 1-aminocyclopropane-1-carboxylate synthase [GenBank:EF151139], hydroperoxide lyase [GenBank:EF151140], glutathione reductase [GenBank:EF633696], 3-oxo-5-alpha-steroid 4-dehydrogenase [GenBank:AY7722579], SKP1 component-like 1 [GenBank:EF624239], amino acid-polyamine transporter [GenBank:EF624240], xanthine dehydrogenase [GenBank:EF624241], auxin influx transport protein [GenBank:EF624242], histidinol dehydrogenase [GenBank:EF624244], phenylalanine ammonia-lyase [GenBank:EF624245], putative auxin efflux carrier protein [GenBank:EF624246], phosphatase 2A 65 kDa regulatory subunit [GenBank:EF624247], sucrose-phosphate synthase [GenBank:EF624248] and sucrose synthase [GenBank:EF624249] a reverse transcriptase–PCR based cloning approach was used. Degenerated primers were designed through alignment of known sequences available at GenBank. The amplicons were cloned using the pGEM cloning kit (Promega, Madison, WI, USA). Allene oxide cyclase [GenBank:AY644677] and allene oxide synthase [GenBank:AY745883] had been previously cloned [8].

### Construction of a cDNA microarray

A hop cDNA library representative of the different morphogenic stages (each morphogenic stage was a pool from 4–6 independent experiments, each one containing dozens of samples) was constructed with ZAP Express cDNA synthesis and ZAP Express cDNA Gigapack III Gold cloning kits (Stratagene, La Jolla, CA, USA). Randomly chosen clones (without prior sequencing) were PCR amplified using T7 and T3 primers and inserts with a length over 400 bp were selected (3,308 cDNAs). cDNAs from the sixteen clones mentioned in previous section as well as an yeast gene (*YAP1*) were included. Amplified cDNA inserts were purified with MultiScreen-PCR plate (Millipore), transferred to printing plates (Microtiter V plates, Sigma), resuspended in 50% dimethylsulphoxide, 0,2% SSC and spotted in duplicate onto Poly-L lysine treated glass slides using VersArray ChipWriter Compact^® ^(Bio-Rad Lab., Hercules, CA, USA). As an additional control, thirty-four clones (previously sequenced) were each printed 4 times on the array in 2 different subgrids. Technical details of the spotting are provided as MIAME (see Additional file 5). After printing, cross-linking was performed with heat and UV according to the protocol from Vodkin laboratory . To examine the quality of the microarrays Gelstar nucleic acid stain (FMC, Rockland, ME) was carried out.

### Target preparation and hybridization

RNA used for hybridizations was obtained from at least 3 independent experiments yielding 3 biological replicates (without mixing RNA samples from the same developmental stage). For each time point at least four hybridizations were carried out corresponding to 3 biological replicates and one dye-swap, except for the time point corresponding to 24 h of culture. This experiment was carried out twice including one dye-swap and it was conducted to evaluate after 24 h of culture the transcript abundance of the differentially expressed genes obtained for the other time points. Three time points (24 h, 15 d, 28 d) were conducted with time 0 as common reference. Additionally another control was used to account for the influence of growth regulators and other culture factors (28 d with growth regulators versus 28 d without growth regulators). Self-self hybridizations were also carried out to evaluate dye bias. Seventy micrograms of RNA from each biological replicate was used for cDNA synthesis with a RT primer (oligo dT) for labelling with either Cy3 or Cy5 dye molecules (Amersham, Buckinghamshire, UK) and Revert Aid Hminus Reverse Transcriptase (Fermentas). Following cDNA synthesis RNA was removed with RNase (Fermentas) and labelled targets purified using QIAquick PCR Purification kit (Quiagen). Prior to hybridization slides were pre-treated in 1% BSA, 5× SSC and 0.1% SDS (w/v) for 30 min at 50°C. Briefly, the hybridization mix consisted of 30 μl of labeled cDNAs, 7.5 μg Cot-1 DNA (Invitrogen), 15 μg Poly (A^+^), 7.5 μg of salmon sperm DNA, 1.5 μl of 50× Denhard't solution, 7.9 μl 20× SSC and 1.5 μl 10% SDS (w/v). Mixture was denatured at 97°C for 2 min, put on ice and then 1 μl of 10× DIG blocking (Roche Diagnostic GmbH, Mannheim, Germany) buffer was added. Hybridization was carried out for 17 h at 65°C in chambers ArrayIt (Telechem International) placed in a water bath. Then slides were washed once in 0.5× SSC and 0.1% SDS (w/v), then in 0.5× SSC and 0.01% SDS (w/v) and twice in 0.06× SSC.

### Signal detection and data analysis

Slides were scanned using VersArray ChipReader^® ^and spot and background intensities quantified using Versarray Analyser software (Bio-Rad). Background was calculated as the trimmed mean of pixel intensity in spots's local corners. Low intensity signal spots (trimmed mean of raw intensity/trimmed mean of background < 1.5), uneven background (trimmed mean of raw intensity/standard deviation of background < 2.5), uneven spots (trimmed mean of raw intensity of background/standard deviation of raw intensity < 1) and spots that are not validated (flags) were removed from analysis before normalization. Data files were imported into GEPAS  and log2-transformed prior to normalization (DNMAD). Print-tip Lowess was used for within-slide normalization considering background subtraction. Statistical analysis was performed using Rank Products (RP) method [23]. RP values, for each gene, were compared to the RPs of 1000 random permutations with the same number of replicates and genes as the real experiment. This rank-based test statistic is a non-parametric method shown to generate accurate results with biological datasets, particularly at small numbers of replicates [68]; it has been already used for analysis of transcriptional profiling in plants [69]. It was considered a FDR < 0.05, and a fold change of 1.87 and -1.87 for up and down-regulation respectively.

### Analysis of sequences and gene annotation

Differentially expressed genes were sequenced and checked for identities (BLASTn and BLASTx) in the database . The E-value threshold was set at 1.0 E^-7^. Larger sequences which did not present Poly (A^+^) were re-sequenced at their 3' ends. Due to cDNA library redundancy which was nearly 45% several clones appeared twice or more. Eighty per cent of the clones which appeared only once were re-sequenced. ESTs were contigged to identify unigenes and each represents the 5'most clone in a contig with maximal base call identity to the contig consensus (Seqman, DNAStar). Clones representative of 133 unigenes were grouped to build a library. Gene annotation was carried out using TAIR . Accession numbers for the sequence data are as follows: [GenBank:AY849555, GenBank:AY795910, GenBank:AY772257, GenBank:AY644677, GenBank:AY745883, GenBank:CD527119, GenBank:CD527120, GenBank:CD527121, GenBank:CD527122, GenBank:CD527123, GenBank:CD527124, GenBank:EF151139, GenBank:EF151140, sequences from GenBank:ES437670 until GenBank:ES437798].

### Quantitative RT-PCR

RNA was sampled as for microarrays analysis. RNA was quantified after DNAse treatment and 7 μg of total RNA for each biological replicate was used to synthesize cDNA separately. Complementary DNAs were then quantified using a spectrofluorimeter (Anthos Zenyth 3100) and brought to equal concentration. qRT-PCR reactions were performed with the Light Cycler Fast Start ReactionMix MasterPLUS SYBR Green I (Roche, Mannheim, Germany) on a Roche light cycler real time PCR machine according to the manufacturer's instructions. The transcript concentration for each sample was calculated based on a standard calibration curve obtained from serial dilutions of plasmid containing the insert to be analysed. A negative control reaction without template was always included for each primer combination. Two-three biological replicates and one technical replicate were performed per time point. The means from 3–4 qRT-PCR reactions are presented for each time point. The following primers were used: for glycolate oxidase-fw 5' CCTCGTATCCTGATTGATGT, rv 5' TGCTGATGCTGCTCTTGCT, amplified fragment 148 bp; for DAHPS-fw 5' CATGTGGTCTCAAGACACG, rv 5' GATCCTCCAATACACTCAGT, amplified fragment 153 bp; for cinnamate 4-hydroxylase-fw 5' GGTGAGAGGAGTAGACTG, rv 5' CTTCAAGAATATGGTCAATGG, amplified fragment 222 bp; for sucrose synthase-fw 5' CCTTCTTGCCCACAAACT, rv 5' GAGTCCAGGAAGAGTGAA, amplified fragment 252 bp; for metallothionein-fw 5' GCAAGTGTGGAAAGAGGTA, rv 5' TTTGTGTGTGTGTGGCTTG, amplified fragment 222 bp; for peroxidase Hl1-fw 5' TGGACTCTACAAGGAGGT, rv 5' TTGTGGTCAGGGAGGTA, amplified fragment 258 bp; and for peroxidase Hl4578-fw 5' GGAAGAAGAGATGGGAGAA, rv 5' TATGGTCGGGTCAGGAAG, amplified fragment 221 bp.

### Metabolic profiling using ^1^H NMR, J-resolved, COSY, HMBC analysis and multivariate analysis

Plant material was frozen and grinded in liquid nitrogen and lyophilized for at least 72 h at -40°C. Twenty five mg of material was used for each sample extraction according essentially to [70]. K_2_PO_4 _was added to D_2_O (99.00%, Cambridge Isotope Laboratories, Miami) as a buffering agent. The pH of the D_2_O for NMR measurements was adjusted to 6.0, using a 1N NaOD solution (Cortec, Paris). Samples were solved in 750 μl of K_2_PO_4 _with 0,1% trimethyl silane propionic acid sodium salt (standard purchased from Merck, Darmstadt, Germany) and 750 μl of methanol-*d*4 (99.8%, Cambridge Isotope Laboratories, Miami). Then, samples were briefly vortexed, sonicated for 10–20 min and centrifuged for 10 min at 13000 rpm. The supernatant (800 μl) was then used for analysis. ^1^H NMR spectra were recorded at 25°C on a 500 MHz Bruker DMX-500 spectrometer operating at a proton NMR frequency of 500.13 MHz. Each ^1^H NMR spectrum consisted of 128 scans requiring 10.26 min measuring time with the following parameters: 0.16 Hz/point, pulse width (PW) = 30°, acquisition time = 3.17 sec, relaxation delay = 1.5 sec. A presaturation sequence was used to suppress the residual water signal at δ 4.91 with low power selective irradiation at the water frequency during the recycle delay. FIDs were Fourier transformed with LB = 0.3 Hz. The resulting spectra were manually phased and baseline corrected, and calibrated to TSP at δ 0.0, all using XWIN NMR (version 3.5, Bruker). The ^1^H NMR spectra were automatically reduced to ASCII files using AMIX (version 3.7, Bruker Biospin). Spectral intensities were scaled to TSP and to total intensity and reduced to integrated regions of equal width (0.04 ppm) corresponding to the region δ = 0.40–10.00. The region of δ = 4.70–5.10 was excluded from the analysis because of the residual signal of water. PCA analysis was carried out with the SIMCA-P software (version 11.0; Umetrics, Umea°, Sweden). The Pareto scaling method was used, which gives each variable a variance numerically equal to its standard deviation.

Excel files containing spectral intensities reduced to integrated regions of equal width (0.04 ppm) were used for Kruskal-Wallis test to evaluate metabolites present in four samples in significantly different amounts (at a *p *value of 0.005). If significant differences existed between the four samples, Wilcoxon rank sum test was applied in order to determine which samples have significantly different amounts. The Kruskal-Wallis test and the Wilcoxon rank sum test are the classical non-parametric alternatives for the ANOVA and for the *t*-test, respectively. Both are more powerful in case of non-normal data.

Two dimensional J-resolved ^1^H-NMR spectra were acquired using 8 scans per 128 increments that were collected into 8 k and 128 data points for F2 and F1 axis, respectively, using spectral widths of 5 kHz in F2 (chemical shift axis) and 66 Hz in F1 (spin-spin coupling constant axis). A 1.49 sec relaxation delay was employed, giving a total acquisition time of 56.7 min. Both dimensions were multiplied by sine-bell functions prior to double complex FT. J-resolved spectra tilted by 45°, symmetrized about F1, and then calibrated, all using XWIN NMR (version 3.5, Bruker).

^1^H-^1^H-correlated spectroscopy (COSY), and heteronuclear multiple bonds coherence (HMBC) spectra were recorded on a 600 MHz Bruker DMX-600 spectrometer (Bruker). The COSY spectra were acquired with 1.0 sec relaxation delay, 6361 Hz spectral width in both dimensions. Window function for COSY spectra was sine-bell (SSB = 0). The HMBC spectra were obtained with 1.0 sec relaxation delay, 6361 Hz spectral width in F2 and 30183 Hz in F1. The optimized coupling constant for HMBC was 8 Hz.

### HPLC Quantification of Thiols

Plant material was frozen in liquid nitrogen, extracted in 0.5M perchloric acid in phosphate buffer saline (PBS) and centrifuged for 5 min at 4°C. Cysteine and glutathione were separated and quantified by HPLC following monobromobimane (Sigma Chemical Co) derivatization of the plant extracts as described by Sousa Silva et al. [71] with minor modifications. Thiol determinations were performed in a Beckman Coulter HPLC coupled to a Jasco FP-2020 Plus fluorescence detector and a Beckman Coulter System Gold 508 auto-sampler. A Merck LichroCART 250-4 (250 × 4 mm) column with stationary phase LiChrospher^® ^100 RP-18 (5 μm) was used. Elution of bimane-derivatized compounds was monitored by fluorescence detection with excitation at 397 nm and emission at 490 nm, using a binary gradient of acetonitrile (HPLC grade, Merck) with 0.08% TFA (solvent A) and water with 0.08% TFA (solvent B). The gradient program was: 0–10 min, 5% solvent B isocratic; 10–35 min, 5–10% solvent B; 35–45 min, 10–30% solvent B; 45–50 min, 30–5% solvent B. Cysteine (Merck) and glutathione (Boehringer Mannheim GmbH) were used as standards. Three-four independent biological replicates were performed per time point.

## Authors' contributions

AMF designed the experiment and wrote the manuscript, performed *in vitro *work, has isolated clones and constructed cDNA microarrays, performed chip hybridizations, has done acquisition, analysis and interpretation of microarray data, sequence analysis, metabolic profiling, and participated in cDNA library construction, gene cloning, sequencing and RT-PCR. FS performed all gene annotation, participated in cDNA library construction, gene cloning, sequencing and sequence analysis. YHC participated in metabolic profiling and in multivariate analysis. MSS participated in cDNA library construction and performed thiols quantification. AF designed primers for RT-PCR and participated in RT-PCR. LS carried out the statistical analysis. FP helped on the use of bioinformatic tools. BA and MS developed protocols for chip construction and microarray hybridization. FS, KP, RM, RV, and MSP critically revised the manuscript. All authors approved the final manuscript.

## Supplementary Material

Additional file 1 **Identities of differentially expressed genes**. Identities were checked using the following databases: National Center for Biotechnology Information-NCBI and The Arabidopsis Information Resource-TAIR. Click here for file

Additional file 2 **Rank Products statistics**. Identification of genes with statistically significant expression changes was carried out using RP test statistics. Click here for file

Additional file 3 Heteronuclear multiple bond correlation (HMBC) corresponding to a T15d sample. Click here for file

Additional file 4 Wilcoxon Rank sum and Kruskal-Wallis statistics. Click here for file

Additional file 5 **Technical Details of Array Design and Spotting**. Technical details of the spotting are provided as MIAME. Click here for file
