# Supplementary figures and images for: Organogenic nodule development in hop (Humulus lupulus L.): Transcript and metabolic responses
Source: BMC Genomics. 2008 Sep 29;9:445. doi: 10.1186/1471-2164-9-445 (PMC2573896; doi:10.1186/1471-2164-9-445)

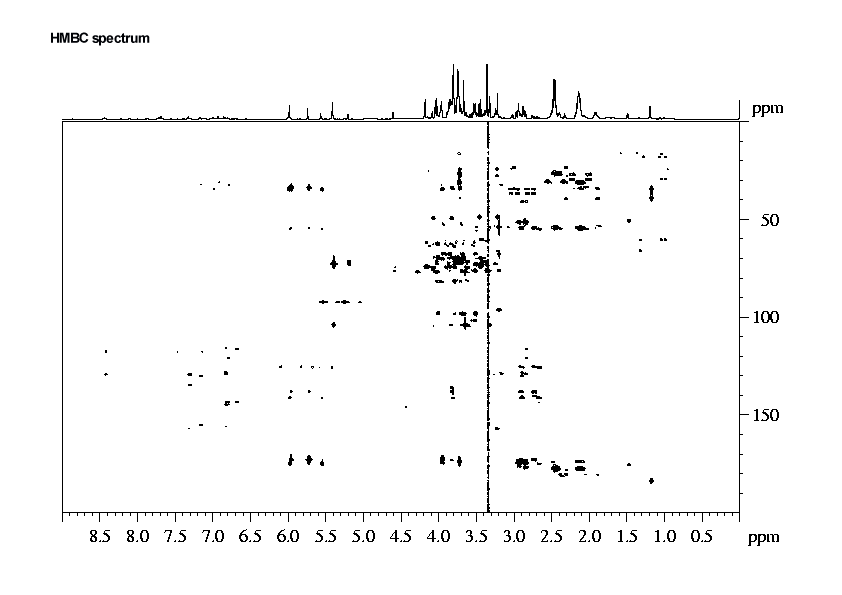

Supplement: Additional file 3 — Heteronuclear multiple bond correlation (HMBC) corresponding to a T15d sample. [file 1471-2164-9-445-S3.tiff]
