# Supplementary material for: Organogenic nodule development in hop (Humulus lupulus L.): Transcript and metabolic responses
Source: BMC Genomics. 2008 Sep 29;9:445. doi: 10.1186/1471-2164-9-445 (PMC2573896; doi:10.1186/1471-2164-9-445)
Supplement: Additional file 5 — Technical Details of Array Design and Spotting. Technical details of the spotting are provided as MIAME. [file 1471-2164-9-445-S5.doc]

**Technical Details of Array Design and Spotting**

Platform type: spotted glass array coated with Poly-L-lysine

Microarrayer: ChipWriter Compact System (Biorad)

Pins: 12 printing pins (Telechem International, Sunnyvale, CA, USA)

Pin configuration: 2 x 6

Number of repeats: spots are repeated once on the same subgrid in the same order (see image below). Last plate contains only printing solution.

Printing plates: 36 x 2 (96 wells, one plate contains only printing solution)

Printing scheme: 12 subgrids, each subgrid with 24 columns and 24 rows (see image below)

Distance between spots: 320μm

Humidity: 40%

Temperature: 22ºC


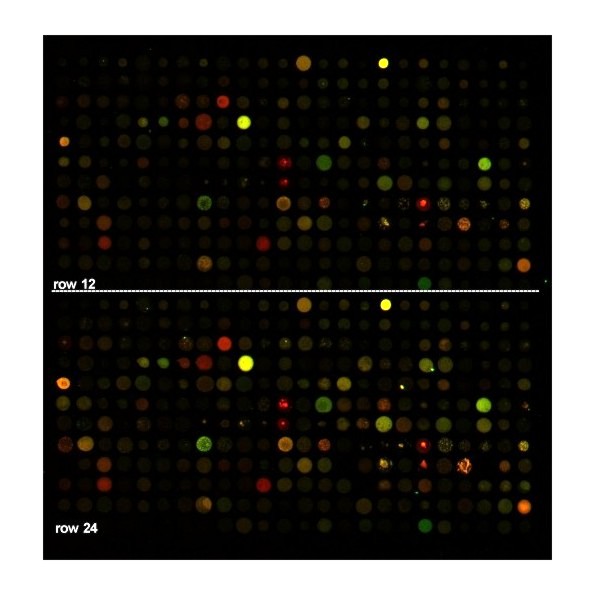
Location of each feature (spot): see supplemental data II

**Figure** One subgrid is depicted. Spots are repeated once on the same subgrid in the same order, thus row 12 represents the same clones as row 24.
